# Predictors of mortality available at emergency department arrival in massively transfused trauma patients without severe TBI: A single-center retrospective cohort study

**DOI:** 10.1371/journal.pone.0359963

**Published:** 2026-10-01

**Authors:** Younggoun Jo, Yunchul Park, Euisung Jeong, Hyunseok Jang, Hyo-Sin Kim

**Affiliations:** Department of Surgery, Chonnam National University Medical School and Hospital, Gwangju, Republic of Korea; Cleveland Clinic, UNITED STATES OF AMERICA

## Abstract

Trauma patients requiring massive transfusion have mortality exceeding 40%, yet early risk-stratification tools for this population remain limited. We evaluated readily available predictors of mortality in massively transfused trauma patients, excluding those with severe traumatic brain injury (TBI) to focus on a hemorrhage-predominant cohort. This single-center retrospective cohort study included trauma patients who received massive transfusion at Chonnam National University Hospital between January 2018 and December 2023. Patients with severe TBI (Head Abbreviated Injury Scale [AIS] ≥ 4) were excluded. Three sequential multivariate logistic regression models were constructed: a primary model using variables available at emergency department (ED) arrival, a secondary model adding laboratory values, and an exploratory model incorporating early transfusion course variables. Of 172 massively transfused trauma patients, 124 met inclusion criteria (Head AIS < 4). Overall mortality was 41.1% (51/124), with 24-hour mortality of 23.7% (28/118). The Glasgow Coma Scale (GCS) was the strongest independent predictor across all models (OR = 0.77–0.79 per point, p < 0.001). In the primary ED arrival model (AUC = 0.770), GCS was the sole significant predictor; GCS alone achieved a comparable AUC of 0.771. The exploratory model incorporating transfusion variables achieved the highest discrimination (AUC = 0.828). All models showed adequate calibration (Hosmer–Lemeshow p > 0.05). Mortality decreased with longer time-to-first-transfusion (54.5% for ≤15 min vs. 23.7% for >60 min, trend p = 0.007), reflecting confounding by indication. In massively transfused trauma patients without severe TBI, GCS assessed at ED arrival is the single most informative predictor of mortality, enabling risk stratification before laboratory results become available. Metabolic acidosis markers and coagulopathy were strongly associated with mortality on univariate analysis but added no independent predictive value beyond GCS. Time-to-first-transfusion appeared to reflect hemorrhage acuity rather than a modifiable prognostic factor.

## Introduction

Hemorrhage remains the leading cause of preventable death in trauma, accounting for approximately 30–40% of trauma mortality [1,2]. Among the most critically injured, those requiring massive transfusion—commonly defined as ≥10 units of red blood cells (RBC) within 24 hours or activation of a massive transfusion protocol (MTP) [3,4]—represent a distinct high-risk population, with reported mortality often exceeding 30%.

The concept of damage control resuscitation, including early balanced blood product administration and permissive hypotension, has fundamentally changed the approach to hemorrhagic shock management [5,6]. Despite these advances, mortality in massively transfused trauma patients remains unacceptably high. Identifying early predictors of mortality is essential for triaging patients, allocating resources, and guiding resuscitation decisions in the emergency department.

Several scoring systems, including the Assessment of Blood Consumption (ABC) score [7] and the Trauma Associated Severe Hemorrhage (TASH) score [8,9], have been developed to predict the need for massive transfusion. However, fewer studies have focused on mortality predictors within the massively transfused population itself, where all patients have already demonstrated severe hemorrhage. Once massive transfusion has been initiated, clinicians need tools for early risk stratification to guide the intensity and goals of ongoing resuscitation.

The physiology recorded at ED arrival is not produced by the injury alone. Fluid and blood products given before arrival modify the first values documented in the ED [5,6], the change in blood pressure and heart rate between the scene and the hospital may carry information that a single arrival reading cannot, and the level of consciousness observed in the field may differ from the value recorded after arrival. Any model anchored at ED arrival therefore describes the patient as encountered at the hospital door, and how much of the preceding phase can be recovered depends on what the trauma system records. We therefore state at the outset what our registry holds: a single set of field vital signs, but no serial prehospital measurements and no record of the volumes given before arrival. This defines what could be modeled here and what could not.

A critical methodological challenge in studying mortality in massively transfused trauma patients is the confounding effect of traumatic brain injury (TBI). Patients with severe TBI (Head Abbreviated Injury Scale [AIS] ≥ 4) have fundamentally different mortality mechanisms—primarily neurological rather than hemorrhagic—and their inclusion may obscure the identification of hemorrhage-related prognostic factors [10]. The Glasgow Coma Scale (GCS), widely used as a trauma severity indicator, has been shown to carry differential prognostic significance depending on the presence or absence of head injury [10].

The purpose of this study was to evaluate readily available clinical predictors of mortality in trauma patients requiring massive transfusion, after excluding those with severe TBI. We specifically sought to determine whether simple bedside parameters available at emergency department (ED) arrival could provide meaningful early risk stratification in this critically ill population.

## Materials and methods

### Study design and setting

This was a single-center retrospective cohort study conducted at Chonnam National University Hospital, a tertiary referral trauma center in Gwangju, Republic of Korea. The study period spanned from January 2018 to December 2023. The study was approved by the Institutional Review Board of Chonnam National University Hospital (IRB No. CNUH-2026–252) and was conducted in accordance with the Declaration of Helsinki. The requirement for informed consent was waived due to the retrospective nature of the study. The trauma registry and electronic medical record data were accessed for research purposes on 16/07/2026 (DD/MM/YYYY). During data collection, the investigators had access to information that could identify individual participants; all data were anonymized prior to analysis.

### Study population

Massively transfused trauma patients during the study period were identified from the institutional trauma registry using predefined criteria: ISS ≥ 9, direct admission (not transferred), and receipt of ≥5 units of packed red blood cells (pRBC) within 4 hours of arrival or ≥10 pRBC units within 24 hours. Patients whose first transfusion commenced after operating room entry were excluded. Patients with severe TBI, defined as Head AIS ≥ 4, were excluded from the primary analysis to focus on a hemorrhage-predominant cohort. The final primary analysis cohort comprised 124 patients (Fig 1).

**Fig 1:**
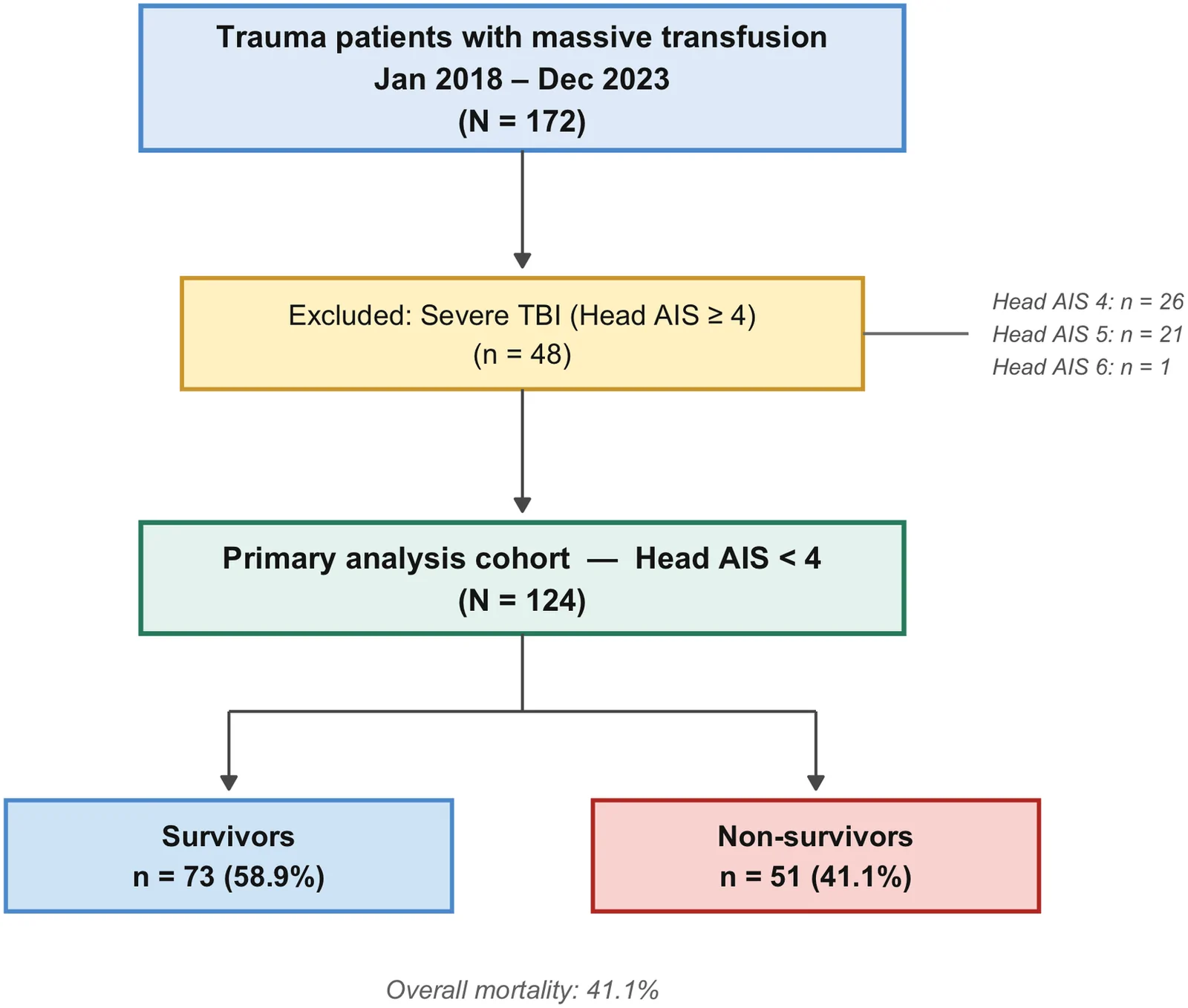
Study flow diagram. Of 172 trauma patients meeting massive transfusion criteria, 48 with severe traumatic brain injury (Head AIS ≥ 4) were excluded. The primary analysis cohort comprised 124 patients. AIS, Abbreviated Injury Scale; TBI, traumatic brain injury; pRBC, packed red blood cells.

### Data collection

Data were extracted from the electronic medical records and trauma registry. Demographic variables included age and sex. Injury characteristics included the Injury Severity Score (ISS), regional AIS scores, and injury mechanism. Physiologic parameters recorded at ED arrival included GCS, systolic blood pressure (SBP), heart rate (HR), and body temperature. Shock index was calculated as HR divided by SBP [18]. SBP was recorded as ‘unmeasurable’ when blood pressure could not be obtained at ED presentation, typically in patients with profound hemorrhagic shock, peri-arrest, or cardiac arrest; this was analyzed as a separate binary variable. Continuous vital-sign readings that were physiologically unobtainable at presentation (e.g., unmeasurable blood pressure or heart rate in peri-arrest patients) were treated as missing for continuous analyses, so that these non-physiologic values did not distort central-tendency estimates or regression coefficients.

Prehospital variables were retrieved from the same registry. Because only patients transported directly from the scene were eligible, a single set of field vital signs documented by emergency medical services—systolic and diastolic blood pressure and heart rate—was potentially available for every patient. Serial prehospital vital-sign measurements, prehospital level of consciousness, and the volumes of fluid or blood products administered before arrival are not captured by the registry and could therefore not be studied. The paired field and ED readings were used to derive the change in systolic blood pressure and heart rate between the scene and hospital arrival, which is the only physiologic trajectory the available data support.

Laboratory values obtained at ED arrival included hemoglobin, arterial lactate, base excess (BE), arterial pH, prothrombin time–international normalized ratio (PT-INR), albumin, and creatinine. Transfusion data included time-to-first-transfusion (TT), defined as the interval from ED arrival to the first RBC unit administered, as well as total RBC units transfused within 4 hours and 24 hours.

### Outcome measures

The primary outcome was in-hospital mortality. The secondary outcome was 24-hour mortality. For the analysis of time-to-transfusion, patients were categorized into four groups: ≤ 15 minutes, 16–30 minutes, 31–60 minutes, and >60 minutes.

### Statistical analysis

Continuous variables were expressed as median (interquartile range [IQR]) and compared using the Wilcoxon rank-sum test. Categorical variables were expressed as number (percentage) and compared using Fisher’s exact test. Univariate logistic regression was performed to identify variables associated with mortality, with results reported as odds ratios (OR) with 95% confidence intervals (CI).

Complete-case analysis was used for all models. Missing data rates were low (≤ 4.8%) for most variables; the principal exception was continuous blood pressure and heart rate, for which physiologically unmeasurable readings in profoundly shocked patients were treated as missing (unmeasurable SBP in 31% and HR in 13% of the cohort) and were instead represented by the unmeasurable-SBP indicator (S1 Table). Mortality rates were comparable between complete and incomplete cases (S2 Table).

Three sequential multivariate logistic regression models were constructed with increasing variable complexity. The primary model included the variables reliably available at ED arrival for every patient: GCS, age, and ISS. Continuous systolic blood pressure and heart rate were not entered into the multivariate models because they were not independently associated with mortality and because physiologically unmeasurable readings in profoundly shocked patients would otherwise have caused substantial case loss; the hemodynamic signal at arrival was instead captured by the unmeasurable-SBP indicator in the univariate analysis. The secondary model added the first available laboratory result: arterial lactate. The exploratory model further incorporated early transfusion course variables: time-to-first-transfusion and RBC units at 4 hours. The primary model was designated as the main analysis because it uses only information available at the point of clinical decision-making; the secondary and exploratory models served to evaluate the incremental discriminative value of laboratory and transfusion variables.

Variable selection for multivariate models was guided by clinical relevance, univariate significance (p < 0.1), and collinearity assessment. Among the metabolic acidosis markers, base excess, arterial pH, and lactate showed high inter-correlation (Spearman |r| = 0.68–0.84; S3 Table). Lactate was selected as the single representative metabolic marker for models including laboratory variables, based on its established role as a resuscitation endpoint and wider clinical availability; base excess and arterial pH were therefore not entered simultaneously. PT-INR was likewise excluded from the multivariate models because its correlation with the metabolic markers and limited sample variation precluded stable estimation alongside lactate. Multicollinearity in the final models was assessed using variance inflation factors (VIF); all VIF values were below 2.2 (S4 Table).

Model discrimination was assessed using the area under the receiver operating characteristic curve (AUC). Model calibration was evaluated using the Hosmer–Lemeshow goodness-of-fit test. The Cochran–Armitage test for trend was used to evaluate the relationship between time-to-transfusion categories and mortality.

Two sets of sensitivity analyses were performed. The first repeated the primary model in the full cohort, including patients with severe TBI, to assess the robustness of the main findings (S5 Table). The second, added after peer review and therefore post hoc, quantified the contribution of prehospital physiology (S6 Table): field systolic blood pressure, field heart rate, field shock index, field hypotension (<90 mmHg), and the field-to-ED change in systolic blood pressure and heart rate were each examined by univariate logistic regression; field systolic blood pressure and the field-to-ED change in systolic blood pressure were then added to the primary model and compared with the same model fitted to the same patients using the likelihood ratio test and the AUC. Field readings of 0 mmHg or 0 beats per minute were treated as unobtainable and handled in the same way as unmeasurable ED readings.

A two-sided p-value < 0.05 was considered statistically significant. Analyses were performed using R (R Foundation for Statistical Computing, Vienna, Austria) and Python 3.11 (statsmodels).

## Results

### Study population

During the study period, 172 trauma patients met the massive transfusion criteria. After excluding 48 patients with severe TBI (Head AIS ≥ 4: AIS 4, n = 26; AIS 5, n = 21; AIS 6, n = 1), 124 patients comprised the primary analysis cohort (Fig 1). The median age was 56 years (IQR 36–66), and 89 patients (71.8%) were male. The most common injury mechanisms were traffic-related (70/124, 56.5%), falls (27/124, 21.8%), and penetrating injuries (13/124, 10.5%). Among the 124 patients, 102 (82.3%) had no head injury (Head AIS = 0) and 22 (17.7%) had mild-to-moderate head injury (Head AIS 1–3).

### Baseline characteristics

The overall mortality was 41.1% (51/124), with 28 deaths (54.9% of non-survivors) occurring within 24 hours. Baseline characteristics stratified by survival status are presented in Table 1. Non-survivors had markedly lower GCS (median 9 vs. 15, p < 0.001) and a higher rate of unmeasurable SBP (43.1% vs. 23.3%, p = 0.030), whereas measured systolic blood pressure did not differ significantly between groups (median 70 vs. 75 mmHg, p = 0.158). Severe chest injury (AIS ≥ 3) was present in 62.9% of the cohort, severe abdominal injury in 46.8%, and severe extremity injury in 49.2%. Hypothermia (<36°C) was documented in 11.3% of patients.

**Table 1:** Baseline characteristics of survivors versus non-survivors among massively transfused trauma patients without severe traumatic brain injury (head AIS < 4, N = 124).

Variable	Overall (N = 124)	Survivors (N = 73)	Non-survivors (N = 51)	p-value
Age (years)	56.0 (35.8, 66.2)	56.0 (37.0, 64.0)	56.0 (35.0, 71.5)	0.603
**Sex**	—	—	—	0.316
Male	89 (72%)	55 (75%)	34 (67%)	—
Female	35 (28%)	18 (25%)	17 (33%)	—
**Injury mechanism**	—	—	—	0.236
Traffic	70 (56%)	46 (63%)	24 (47%)	—
Fall	27 (22%)	12 (16%)	15 (29%)	—
Penetrating	13 (10%)	8 (11%)	5 (10%)	—
Other	14 (11%)	7 (10%)	7 (14%)	—
**GCS**	14.0 (7.8, 15.0)	15.0 (14.0, 15.0)	9.0 (3.0, 14.0)	**<0.001**
ISS	23.0 (16.0, 29.0)	22.0 (16.0, 29.0)	24.0 (16.0, 27.0)	0.583
**Head AIS 1–3 (mild–moderate)**	—	—	—	0.093
No	102 (82%)	64 (88%)	38 (75%)	—
Yes	22 (18%)	9 (12%)	13 (25%)	—
**Chest AIS ≥ 3**	—	—	—	0.089
No	46 (37%)	32 (44%)	14 (27%)	—
Yes	78 (63%)	41 (56%)	37 (73%)	—
**Abdomen AIS ≥ 3**	—	—	—	0.855
No	66 (53%)	38 (52%)	28 (55%)	—
Yes	58 (47%)	35 (48%)	23 (45%)	—
**Extremity AIS ≥ 3**	—	—	—	0.148
No	63 (51%)	33 (45%)	30 (59%)	—
Yes	61 (49%)	40 (55%)	21 (41%)	—
Systolic BP (mmHg)	70.0 (60.0, 90.0)	75.0 (60.0, 100.0)	70.0 (60.0, 80.0)	0.158
Heart Rate (bpm)	100.0 (84.8, 120.0)	107.0 (87.0, 120.5)	92.0 (84.2, 110.5)	0.181
Body Temperature (°C)	36.1 (36.0, 36.4)	36.1 (36.0, 36.4)	36.0 (36.0, 36.3)	0.165
**Hypothermia (<36°C)**	—	—	—	0.777
No	110 (89%)	64 (88%)	46 (90%)	—
Yes	14 (11%)	9 (12%)	5 (10%)	—
**SBP unmeasurable**	—	—	—	**0.030**
No	85 (69%)	56 (77%)	29 (57%)	—
Yes	39 (31%)	17 (23%)	22 (43%)	—
**Time-to-Transfusion (min)**	34.0 (18.0, 75.0)	45.0 (23.0, 101.0)	23.0 (16.0, 43.0)	**0.002**
**RBC units at 4 h**	6.0 (5.0, 10.0)	6.0 (5.0, 8.0)	7.0 (6.0, 12.5)	**0.029**
**RBC units total**	11.0 (7.0, 15.0)	10.0 (6.0, 13.0)	13.0 (10.0, 18.5)	**0.002**
**Massive transfusion (>10 U/24 h)**	—	—	—	0.258
No	99 (80%)	61 (84%)	38 (75%)	—
Yes	25 (20%)	12 (16%)	13 (25%)	—
Hemoglobin (g/dL)	11.1 (9.0, 12.4)	11.3 (9.5, 12.5)	10.4 (8.8, 12.4)	0.226
**Lactate (mmol/L)**	5.8 (3.7, 9.0)	5.0 (3.0, 7.1)	8.0 (5.1, 13.9)	**<0.001**
**Base Excess (mEq/L)**	−8.3 (−15.7, −5.2)	−6.7 (−11.6, −3.7)	−12.7 (−19.2, −7.0)	**<0.001**
**Arterial pH**	7.3 (7.2, 7.4)	7.3 (7.3, 7.4)	7.3 (7.1, 7.3)	**<0.001**
**PT-INR**	1.2 (1.1, 1.4)	1.1 (1.1, 1.2)	1.3 (1.2, 1.6)	**<0.001**
Albumin (g/dL)	3.2 (2.8, 3.5)	3.2 (2.9, 3.6)	3.1 (2.7, 3.4)	0.284
Creatinine (mg/dL)	1.0 (0.8, 1.3)	1.0 (0.8, 1.2)	1.1 (0.8, 1.3)	0.841

Continuous variables are presented as median (interquartile range) and categorical variables as n (%). Systolic blood pressure, heart rate, and body temperature exclude physiologically unmeasurable readings (recoded as missing); unmeasurable systolic blood pressure is reported separately as a categorical variable. P values were calculated with the Mann–Whitney U test for continuous variables and the Fisher exact test for categorical variables; bold denotes p < 0.05. Missing values: GCS n = 4; Systolic BP n = 39; Heart Rate n = 16; Body Temperature n = 21; Lactate n = 2; Base Excess n = 2; Arterial pH n = 2; Albumin n = 1; Creatinine n = 1. AIS, Abbreviated Injury Scale; GCS, Glasgow Coma Scale; ISS, Injury Severity Score; PT-INR, prothrombin time–international normalized ratio; RBC, red blood cell.

Non-survivors demonstrated significantly more deranged metabolic parameters, including higher lactate (8.0 vs. 5.0 mmol/L, p < 0.001), more negative base excess (−12.7 vs. −6.7 mEq/L, p < 0.001), lower pH (7.26 vs. 7.34, p < 0.001), and higher PT-INR (1.3 vs. 1.1, p < 0.001). Non-survivors also had shorter time-to-first-transfusion (23 vs. 45 min, p = 0.002) and received more RBC units at 4 hours (7 vs. 6 units, p = 0.029) and in total (13 vs. 10 units, p = 0.002). The distributions of these variables by survival status are shown in Fig 2.

**Fig 2:**
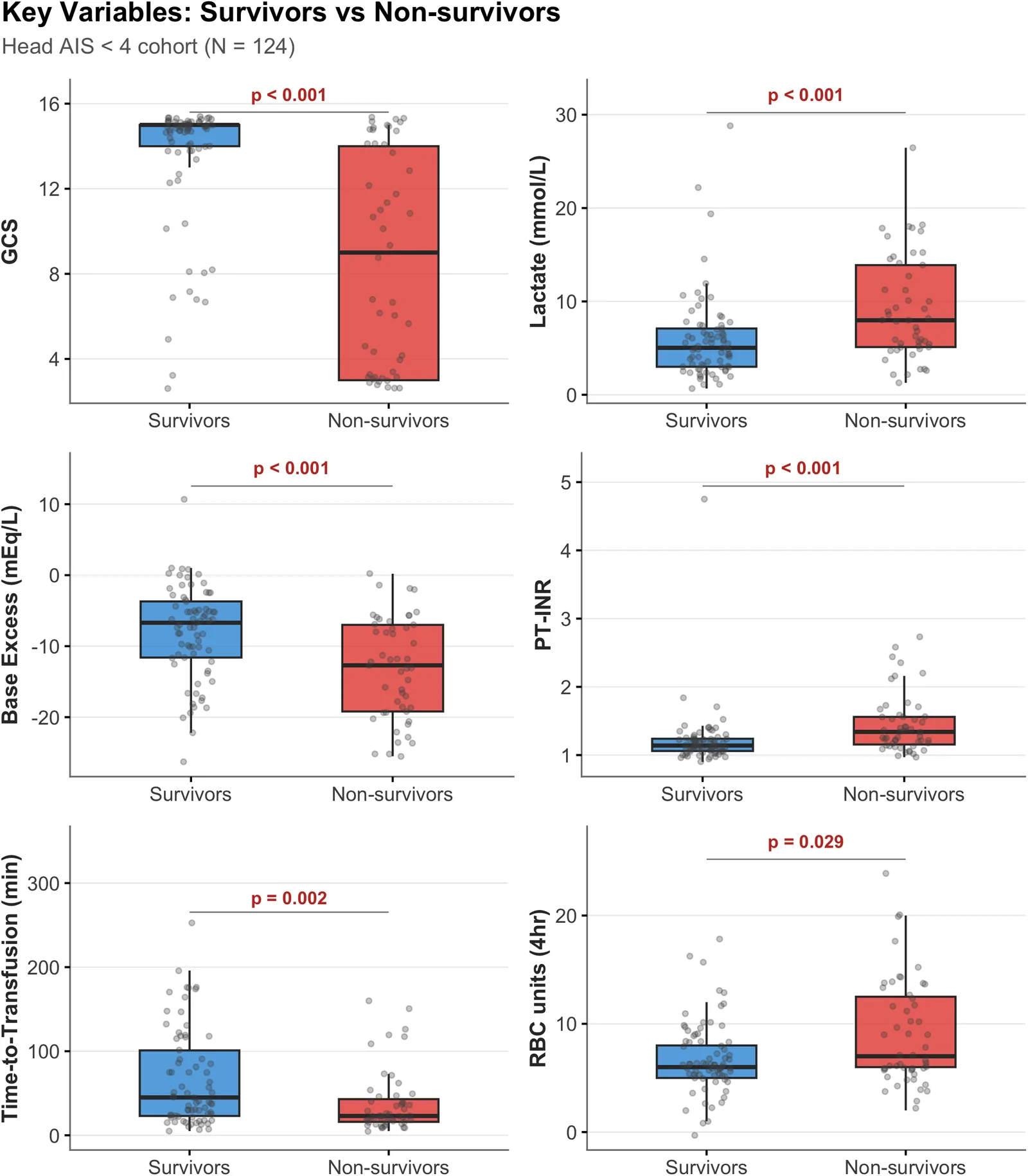
Key variables compared between survivors and non-survivors. Boxplots with individual data points for GCS, lactate, base excess, PT-INR, time-to-transfusion, and RBC units at 4 hours. Non-survivors had significantly lower GCS, higher lactate, more negative base excess, higher PT-INR, shorter time-to-transfusion, and greater RBC consumption. P-values from Wilcoxon rank-sum test; bold red indicates p < 0.05. GCS, Glasgow Coma Scale; PT-INR, prothrombin time–international normalized ratio; RBC, red blood cell.

### Univariate analysis

Univariate logistic regression identified multiple significant predictors of mortality (Table 2). GCS demonstrated the strongest association (OR = 0.78 per point increase, 95% CI: 0.71–0.86, p < 0.001). Other significant predictors included time-to-transfusion (OR = 0.987 per minute, p = 0.005), RBC units at 4 hours (OR = 1.14, p = 0.007), total RBC units (OR = 1.09, p = 0.003), and unmeasurable SBP (OR = 2.50, p = 0.021). Metabolic and coagulation markers were all highly significant: lactate (OR = 1.13, p = 0.002), base excess (OR = 0.90, p < 0.001), pH (OR < 0.01, p < 0.001), and PT-INR (OR = 4.94, p = 0.012). In contrast, measured systolic blood pressure (OR = 0.98, p = 0.144) and heart rate (OR = 0.99, p = 0.216) were not significantly associated with mortality; their apparent association disappeared once physiologically unmeasurable readings were excluded from the continuous analysis.

**Table 2:** Univariate logistic regression for in-hospital mortality (head AIS < 4, N = 124).

Variable	N	OR	95% CI	p-value
Age (per year)	124	1.00	0.99–1.02	0.679
Sex (Female vs Male)	124	1.53	0.69–3.36	0.292
**GCS (per 1-point increase)**	120	0.78	0.71–0.86	**<0.001**
ISS (per 1-point increase)	124	1.01	0.98–1.05	0.543
SBP (per mmHg)	85	0.98	0.96–1.01	0.144
Heart Rate (per bpm)	108	0.99	0.97–1.01	0.216
**Time-to-Transfusion (per min)**	124	0.99	0.98–1.00	**0.005**
**RBC units at 4 h**	124	1.14	1.04–1.25	**0.007**
**RBC units total**	124	1.09	1.03–1.16	**0.003**
Hemoglobin (g/dL)	124	0.90	0.77–1.05	0.184
**Lactate (per mmol/L)**	122	1.13	1.05–1.23	**0.002**
**Base Excess (per mEq/L)**	122	0.90	0.85–0.95	**<0.001**
**Arterial pH (per unit)**	122	0.00	0.00–0.05	**<0.001**
**PT-INR (per unit)**	124	4.94	1.42–17.19	**0.012**
Albumin (per g/dL)	123	0.65	0.34–1.27	0.210
Creatinine (per mg/dL)	123	1.03	0.50–2.12	0.946
**SBP unmeasurable (Yes vs No)**	124	2.50	1.15–5.43	**0.021**
Massive transfusion (Yes vs No)	124	1.74	0.72–4.21	0.219

N denotes the number of patients with the variable available. Odds ratios are per one-unit increase unless otherwise indicated. Bold denotes p < 0.05. CI, confidence interval; GCS, Glasgow Coma Scale; ISS, Injury Severity Score; OR, odds ratio; PT-INR, prothrombin time–international normalized ratio; RBC, red blood cell; SBP, systolic blood pressure.

### Multivariate analysis

Three sequential multivariate models were constructed (Table 3). In the primary model (ED arrival variables only, n = 120), GCS was the sole independent predictor of mortality (OR = 0.776, 95% CI: 0.702–0.859, p < 0.001; AUC = 0.770; Fig 3). Neither age nor ISS reached statistical significance after adjustment. The Hosmer–Lemeshow test confirmed adequate calibration (p = 0.831).

**Table 3:** Sequential multivariate logistic regression models for in-hospital mortality (head AIS < 4). The primary model uses variables reliably available at emergency department arrival (GCS, age, ISS); the secondary model adds the first laboratory result (lactate); the exploratory model adds early resuscitation-course variables (time-to-transfusion, RBC units at 4 h).

Variable	OR	95% CI	p-value
***Primary: ED arrival (N = 120)***			
**GCS**	0.776	0.702–0.859	**<0.001**
Age	1.010	0.988–1.033	0.375
ISS	1.004	0.963–1.048	0.842
**AUC (95% CI)**	**0.770**	**0.677–0.863**	—
Hosmer–Lemeshow p	—	—	0.831
***Secondary: + laboratory (N = 118)***			
**GCS**	0.786	0.703–0.879	**<0.001**
Age	1.015	0.991–1.039	0.224
ISS	1.007	0.964–1.051	0.759
Lactate	1.038	0.946–1.140	0.428
**AUC (95% CI)**	**0.786**	**0.698–0.875**	—
Hosmer–Lemeshow p	—	—	0.617
***Exploratory: + transfusion (N = 118)***			
**GCS**	0.768	0.679–0.868	**<0.001**
Age	1.023	0.997–1.050	0.087
ISS	0.995	0.950–1.042	0.833
Lactate	1.005	0.911–1.108	0.927
**Time-to-Transfusion**	0.987	0.976–0.999	**0.028**
RBC units at 4 h	1.089	0.968–1.224	0.155
**AUC (95% CI)**	**0.828**	**0.749–0.907**	—
Hosmer–Lemeshow p	—	—	0.802

Odds ratios are per one-unit increase. A Hosmer–Lemeshow p > 0.05 indicates adequate calibration. Bold denotes p < 0.05. Continuous systolic blood pressure and heart rate were not retained because they were not independently associated with mortality and their inclusion caused substantial case loss from physiologically unmeasurable readings; the hemodynamic signal at arrival is captured by the unmeasurable-systolic-blood-pressure indicator (Table 2). Base excess, arterial pH, and PT-INR were excluded because of high collinearity with lactate (Spearman |r| up to 0.84); lactate was retained as the most clinically actionable metabolic marker. AUC, area under the receiver operating characteristic curve; GCS, Glasgow Coma Scale; ISS, Injury Severity Score; OR, odds ratio; RBC, red blood cell.

**Fig 3:**
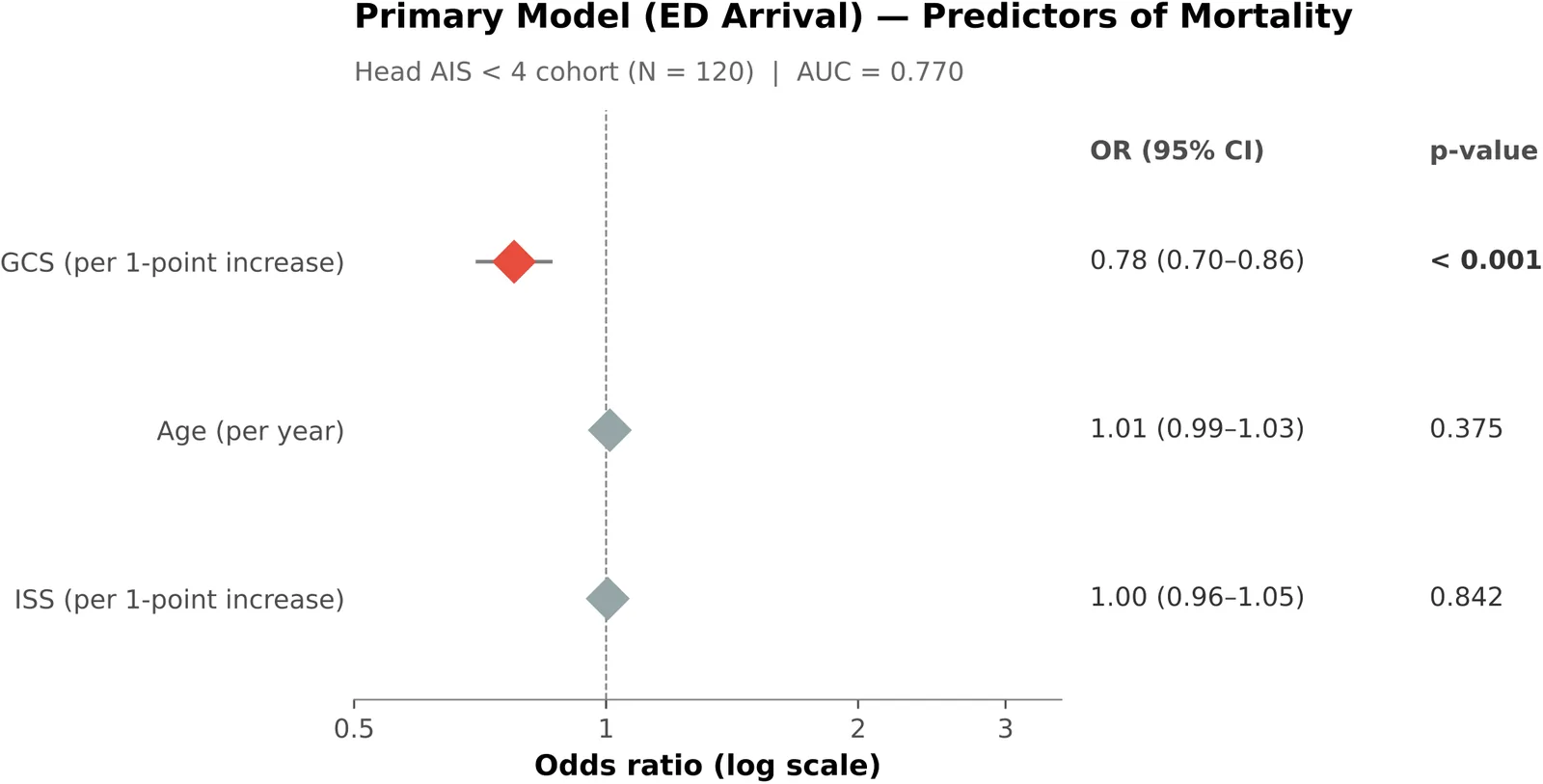
Forest plot of the primary multivariate logistic regression model (ED arrival variables: GCS, age, and Injury Severity Score) for mortality prediction in trauma patients requiring massive transfusion (Head AIS < 4, N = 120). The Glasgow Coma Scale was the only statistically significant independent predictor. The red diamond indicates statistical significance (p < 0.05). OR, odds ratio; CI, confidence interval; GCS, Glasgow Coma Scale; ISS, Injury Severity Score; AUC, area under the curve.

In the secondary model (+ laboratory, n = 118), adding arterial lactate marginally improved discrimination (AUC = 0.786). GCS remained the only significant predictor (OR = 0.786, 95% CI: 0.703–0.879, p < 0.001), while lactate was not independently associated with mortality (OR = 1.04, p = 0.428), consistent with the shared information content among GCS and the metabolic markers (S3 and S4 Tables).

The exploratory model (+ transfusion course, n = 118) incorporated time-to-transfusion and RBC units at 4 hours, achieving the highest discrimination (AUC = 0.828; Fig 4). GCS remained the dominant predictor (OR = 0.768, 95% CI: 0.679–0.868, p < 0.001). Time-to-transfusion was independently associated with mortality in this model (OR = 0.987 per minute, 95% CI: 0.976–0.999, p = 0.028), and age approached significance (OR = 1.023, p = 0.087). All three models demonstrated adequate calibration by the Hosmer–Lemeshow test (p > 0.05), though this test has limited power in samples of this size.

**Fig 4:**
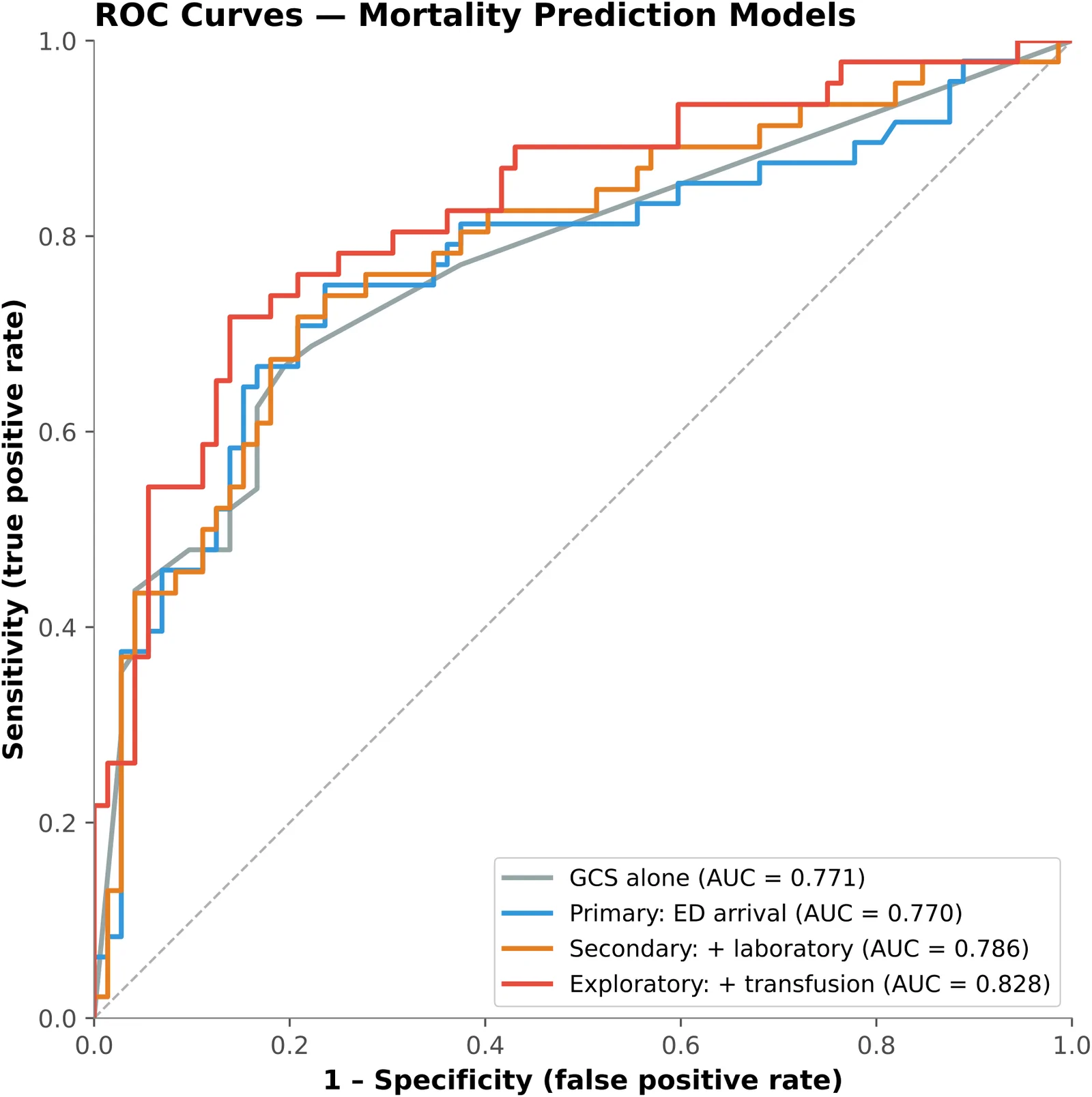
Receiver operating characteristic (ROC) curves comparing four mortality prediction models. GCS alone achieved an AUC of 0.771. Sequential addition of bedside parameters (primary model: AUC = 0.770), laboratory values (secondary model: AUC = 0.786), and transfusion variables (exploratory model: AUC = 0.828) provided incremental improvement. AUC, area under the curve; GCS, Glasgow Coma Scale.

The discriminative performance of GCS alone (AUC = 0.771) was essentially identical to the primary ED arrival model (AUC = 0.770), indicating that GCS contributes virtually all of the predictive power available at arrival. The incremental benefit of laboratory and transfusion variables brought the AUC from 0.771 to 0.828 (Fig 4), a clinically modest improvement.

### Time-to-first-transfusion and mortality

A significant inverse dose-response relationship was observed between time-to-first-transfusion and mortality (trend p = 0.007; Fig 5). Mortality was highest in patients receiving their first RBC unit within 15 minutes of ED arrival (54.5%, 12/22) and progressively decreased through the 16–30-minute (51.4%, 19/37), 31–60-minute (40.7%, 11/27), and >60-minute (23.7%, 9/38) categories. This pattern reflects the clinical reality that patients requiring the most urgent transfusion are those with the most severe hemorrhage.

**Fig 5:**
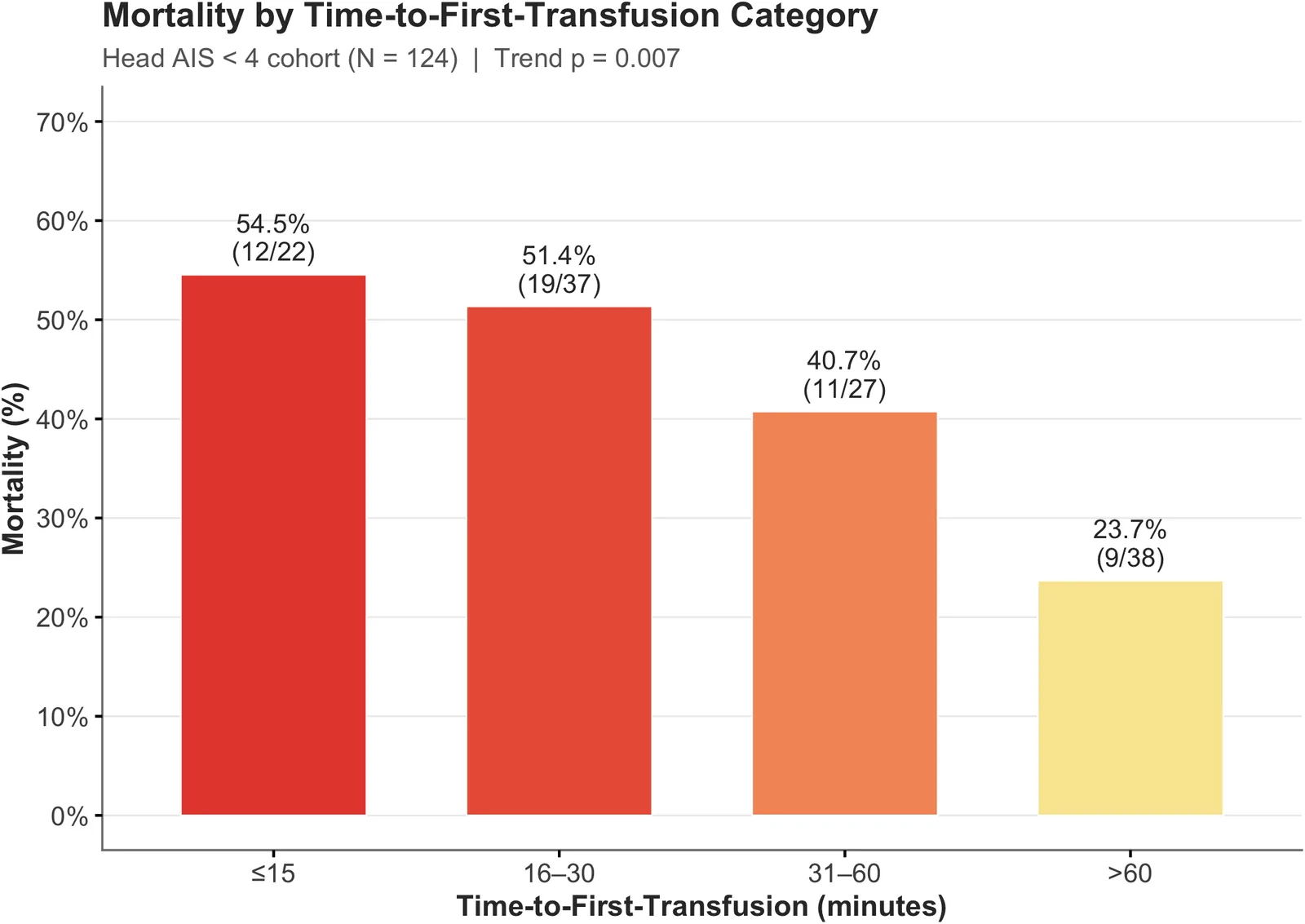
Mortality by time-to-first-transfusion category. Mortality decreased progressively with longer time-to-first-transfusion (≤15 min: 54.5%; 16–30 min: 51.4%; 31–60 min: 40.7%; > 60 min: 23.7%; trend p = 0.007), reflecting confounding by indication—patients requiring the most urgent transfusion had the highest hemorrhage acuity.

### Prehospital physiology and field-to-ED change in vital signs

A field systolic blood pressure was documented in 106 of the 124 patients (85.5%) and was measurable in 103 (83.1%); a field heart rate was documented in 121 (97.6%) and was measurable in 118 (95.2%). A measurable field systolic pressure was thus available in more patients than a measurable systolic pressure at ED arrival (103 vs. 85). Field systolic blood pressure was 80 mmHg (IQR 70–100) in survivors and 80 mmHg (IQR 70–90) in non-survivors (p = 0.330), and field heart rate was 92 bpm (IQR 80–111) versus 98 bpm (IQR 80–127) (p = 0.572).

None of the prehospital measures was associated with in-hospital mortality: field systolic blood pressure (OR = 0.98 per 10 mmHg, 95% CI: 0.84–1.14, p = 0.778), field heart rate (OR = 1.09 per 10 bpm, 95% CI: 0.96–1.24, p = 0.164), field shock index (OR = 1.06 per 0.1, 95% CI: 0.97–1.15, p = 0.215), and field hypotension below 90 mmHg (OR = 1.13, 95% CI: 0.50–2.52, p = 0.774). The change between the field and ED readings—the only physiologic trajectory the registry supports—was likewise unassociated with mortality, for systolic blood pressure (OR = 0.88 per 10 mmHg, 95% CI: 0.73–1.05, p = 0.153; n = 75) and for heart rate (OR = 0.90 per 10 bpm, 95% CI: 0.77–1.05, p = 0.191; n = 103). Adding field systolic blood pressure to the primary model left GCS unchanged (OR = 0.77, 95% CI: 0.68–0.86, p < 0.001), and neither model fit (likelihood ratio p = 0.610) nor discrimination improved beyond the same model fitted to the same 101 patients (AUC 0.792 vs. 0.780). These analyses were post hoc and are reported in full in S6 Table.

### 24-hour mortality

Among 118 patients with available 24-hour mortality data (6 missing; S1 Table), 28 (23.7%) died within 24 hours. In multivariate analysis (n = 114), GCS was the only independent predictor of 24-hour mortality (OR = 0.805, 95% CI: 0.730–0.889, p < 0.001; AUC = 0.760). Neither age nor ISS reached significance for early mortality (S5 Table).

### Sensitivity analysis

When the analysis was repeated using the full cohort including severe TBI patients (n = 165 with complete data), GCS remained the strongest independent predictor of mortality in the primary model (OR = 0.758, p < 0.001; AUC = 0.822). Age showed a borderline association (OR = 1.02, p = 0.053), likely reflecting the poor prognosis of elderly patients with severe TBI. The consistency of GCS as the dominant predictor across both cohorts supports the robustness of our findings (S5 Table).

## Discussion

In this single-center retrospective study of 124 trauma patients requiring massive transfusion without severe TBI, GCS assessed at ED arrival emerged as the single most informative predictor of mortality. A primary model using only bedside parameters reliably available at arrival (GCS, age, ISS) achieved an AUC of 0.770, and GCS alone achieved 0.771—demonstrating that meaningful risk stratification is possible at the moment of clinical decision-making, before laboratory results or transfusion data become available.

The finding that GCS is a powerful mortality predictor even after excluding severe TBI is clinically relevant. In this cohort, 82.3% of patients had no head injury (Head AIS = 0), suggesting that depressed GCS in this population likely reflects the severity of systemic physiologic derangement rather than primary neurological injury. Osler et al. demonstrated that GCS carries differential prognostic significance depending on the presence of head injury, with GCS in non-head-injured patients reflecting systemic physiologic compromise [10]. Our findings are consistent with this concept in the massively transfused population. Importantly, GCS in this context should be understood as a pragmatic composite bedside marker that integrates multiple physiologic inputs—including perfusion status, metabolic derangement, and potentially sedation or intubation effects—rather than a direct measure of any single pathophysiologic mechanism. Whether the link between depressed GCS and mortality operates through cerebral hypoperfusion, global metabolic derangement, or treatment-related factors cannot be established from our observational data.

Because the model is anchored at ED arrival, the prehospital phase deserves explicit consideration rather than a passing mention. Transfusion and fluid given before arrival can move the first values documented in the ED, and a trajectory of deteriorating or improving vital signs may carry information that a single reading cannot. In this cohort the field measurements were well documented—a measurable field systolic pressure was available in more patients than a measurable pressure at arrival—yet neither the field values, the field shock index, nor the field-to-ED change in blood pressure or heart rate was associated with mortality, and adding field systolic pressure to the arrival model neither displaced GCS nor improved discrimination (S6 Table). This mirrors the behavior of the ED hemodynamic variables in the same patients and sharpens the central observation: among patients already committed to massive transfusion, blood pressure and heart rate—whether measured in the field, at arrival, or as the change between the two—separate survivors from non-survivors far less well than the level of consciousness does. What these data cannot address is prehospital treatment itself. The volumes of fluid and blood products given before arrival are not recorded in the registry, and prehospital level of consciousness is not captured, so the extent to which prehospital care shapes the arrival profile could not be estimated. Serial field vital signs, prehospital treatment volumes, and field GCS should be collected prospectively before the incremental value of a GCS-centered arrival model can be considered settled.

The strong univariate associations of lactate (OR = 1.13), base excess (OR = 0.90), pH (OR < 0.01), and PT-INR (OR = 4.94) with mortality are consistent with the well-established role of metabolic acidosis and coagulopathy in the “lethal triad” of trauma—hypothermia, acidosis, and coagulopathy [11]. Qi et al. reported that base excess, lactate, and pH all independently predicted 72-hour mortality in multiply injured patients [12], while Odom et al. demonstrated that lactate clearance is a strong predictor of trauma mortality [13]. That these markers lost significance in multivariate analysis in our study reflects shared information content among GCS, lactate, and the acid-base parameters (Spearman |r| = 0.43–0.84; S3 Table), rather than a lack of clinical importance. BE, pH, and lactate were highly inter-correlated (|r| = 0.68–0.84), precluding their simultaneous inclusion in multivariate models without introducing problematic collinearity.

Early coagulopathy, reflected by PT-INR, was a significant univariate predictor (OR = 4.94, p = 0.012). This aligns with the landmark study by MacLeod et al. showing that early coagulopathy predicts mortality in trauma [14], and with Peltan et al.’s finding that INR-defined acute traumatic coagulopathy is associated with mortality and organ failure [15]. Although PT-INR was not included in the multivariate models due to correlation with the metabolic markers and limited distributional variation in this sample, its strong univariate association underscores the importance of early coagulation monitoring and targeted replacement in massively transfused patients.

An important finding of this study is the inverse relationship between time-to-first-transfusion and mortality (trend p = 0.007). Rather than indicating that early transfusion is harmful, this reflects confounding by indication: patients receiving transfusion within 15 minutes are those with the most severe, immediately life-threatening hemorrhage. This finding is consistent with a recent JAMA Surgery study by Torres et al. examining whole blood transfusion timing in severe hemorrhage [16], where the relationship between transfusion timing and outcomes was modulated by hemorrhage severity. In this observational cohort, time-to-first-transfusion thus appeared to function as a surrogate marker of hemorrhage acuity rather than a modifiable prognostic factor. It remained independently associated with mortality in the exploratory model, but that association is most plausibly explained by confounding by indication, which is why the variable was confined to the exploratory model rather than the primary prediction model.

Our findings have practical implications for trauma resuscitation. First, GCS assessed at ED arrival—a single, universally available parameter—provides the most informative early prognostic signal in massively transfused trauma patients without severe TBI. This suggests that complex multiparameter scoring may not substantially improve upon GCS-centered risk assessment in this specific population. Second, although laboratory markers (lactate, base excess, pH, PT-INR) did not add independent predictive value in multivariate models, their strong univariate associations and established physiologic significance support their continued use as resuscitation targets and monitoring endpoints. Third, the designation of time-to-transfusion as a confounded variable rather than a causal predictor is important for the correct interpretation of transfusion timing data in observational studies.

Our findings align with those of Barbosa et al., who developed a predictive model for mortality in massively transfused trauma patients and identified GCS as a key predictor [17]. However, our study extends this work by explicitly excluding severe TBI and demonstrating that GCS retains its predictive power in a hemorrhage-predominant cohort, and by quantifying the limited incremental value of laboratory and transfusion variables beyond bedside assessment.

### Limitations

This study has several limitations. Its single-center, retrospective design limits generalizability and is susceptible to selection bias. The cohort of 124 patients, while typical for single-center massive transfusion series, limits the number of variables that can be entered reliably into multivariate models and the power to detect modest effects; because no external validation was performed, the models should be read as a description of this population rather than as tools ready for use elsewhere. Although patients with severe TBI (Head AIS ≥ 4) were excluded, 17.7% of the cohort had mild-to-moderate head injury (Head AIS 1–3), so the cohort represents patients without severe TBI rather than a purely hemorrhagic population, and residual confounding from head injury cannot be excluded. Time-to-transfusion was measured from ED arrival, and, as discussed above, prehospital transfusion and fluid volumes, serial field vital signs, and field GCS were not recorded in the registry. Tranexamic acid administration and intubation status at ED arrival were likewise unavailable, and both may influence GCS as well as outcome. Complete-case analysis was used: missing data rates were low (≤ 4.8%) for most variables, but physiologically unmeasurable blood pressure and heart rate (unmeasurable SBP in 31% of patients) reduced the effective sample for those continuous parameters, which is why the hemodynamic signal at arrival was carried by the unmeasurable-SBP indicator rather than by continuous pressure; mortality among complete cases was otherwise similar to that of the cohort as a whole (S1 and S2 Tables).

## Conclusion

In trauma patients requiring massive transfusion without severe TBI, the Glasgow Coma Scale is the single most informative predictor of mortality, enabling early risk stratification at the point of ED arrival. A bedside model centered on GCS achieves discrimination comparable to more complex models incorporating laboratory and transfusion variables, and neither prehospital nor ED hemodynamic variables added to it. Metabolic acidosis markers and early coagulopathy, while strongly associated with mortality, share substantial prognostic information with GCS and retain value primarily as resuscitation endpoints rather than independent predictors. Time-to-first-transfusion appeared to reflect hemorrhage acuity rather than functioning as a modifiable factor. External validation of GCS-based risk stratification in massively transfused trauma patients is warranted.

## Supporting information

S1 Table Missing data summary. (DOCX)

S2 Table Complete-case availability by model. (DOCX)

S3 Table Spearman correlation matrix of key predictor variables. (DOCX)

S4 Table Variance inflation factors for the sequential multivariate models. (DOCX)

S5 Table Sensitivity analyses: 24-hour mortality and the full cohort including severe traumatic brain injury. (DOCX)

S6 Table Post hoc analysis of prehospital physiology and of the field-to-emergency-department change in vital signs. (DOCX)

S1 Checklist STROBE checklist for reporting this retrospective cohort study. (DOCX)
