# Supplementary material for: Predictors of mortality available at emergency department arrival in massively transfused trauma patients without severe TBI: A single-center retrospective cohort study
Source: PLoS One. 2026 Oct 1;21(10):e0359963. doi: 10.1371/journal.pone.0359963 (PMC13630199; doi:10.1371/journal.pone.0359963)
Supplement: Supplementary file 1 [file pone.0359963.s001.docx]

**S1 Table.** Missing data summary.

| **Variable** | **Available** | **Missing** | **% Missing** |
| --- | --- | --- | --- |
| GCS | 120 | 4 | 3.2 |
| Age | 124 | 0 | 0.0 |
| ISS | 124 | 0 | 0.0 |
| SBP | 85 | 39 | 31.5 |
| Heart Rate | 108 | 16 | 12.9 |
| Lactate | 122 | 2 | 1.6 |
| Base Excess | 122 | 2 | 1.6 |
| Arterial pH | 122 | 2 | 1.6 |
| PT-INR | 124 | 0 | 0.0 |
| Hemoglobin | 124 | 0 | 0.0 |
| Time-to-Transfusion | 124 | 0 | 0.0 |
| RBC units at 4 h | 124 | 0 | 0.0 |
| 24-hour mortality | 118 | 6 | 4.8 |

Missing data were minimal for most variables. The exceptions were continuous systolic blood pressure and heart rate: physiologically unmeasurable readings in profoundly shocked patients were treated as missing for the continuous analyses and were instead represented by the unmeasurable-systolic-blood-pressure indicator (analyzed as a categorical variable).
