# Supplementary material for: Predictors of mortality available at emergency department arrival in massively transfused trauma patients without severe TBI: A single-center retrospective cohort study
Source: PLoS One. 2026 Oct 1;21(10):e0359963. doi: 10.1371/journal.pone.0359963 (PMC13630199; doi:10.1371/journal.pone.0359963)
Supplement: Supplementary file 2 [file pone.0359963.s002.docx]

**S2 Table.** Complete-case availability by model.

| **Model** | **N** | **% of cohort** | **Mortality (%)** | **Primary missing variable** |
| --- | --- | --- | --- | --- |
| Primary (ED arrival: GCS, age, ISS) | 120 | 96.8 | 40.0 | GCS missing (n=4) |
| Secondary (+ lactate) | 118 | 95.2 | 39.0 | GCS or lactate missing |
| Exploratory (+ transfusion) | 118 | 95.2 | 39.0 | GCS, lactate, or transfusion missing |

Mortality rates were similar between complete and incomplete cases, supporting the missing-at-random assumption for the complete-case analysis.
