# Supplementary material for: Predictors of mortality available at emergency department arrival in massively transfused trauma patients without severe TBI: A single-center retrospective cohort study
Source: PLoS One. 2026 Oct 1;21(10):e0359963. doi: 10.1371/journal.pone.0359963 (PMC13630199; doi:10.1371/journal.pone.0359963)
Supplement: Supplementary file 3 [file pone.0359963.s003.docx]

**S3 Table.** Spearman correlation matrix of key predictor variables.

| **Variable** | **GCS** | **Lactate** | **Base Excess** | **pH** | **PT-INR** | **SBP** | **Heart Rate** |
| --- | --- | --- | --- | --- | --- | --- | --- |
| GCS | 1.00 | -0.43 | 0.45 | 0.45 | -0.37 | 0.28 | -0.10 |
| Lactate | -0.43 | 1.00 | -0.83 | -0.68 | 0.54 | -0.47 | 0.31 |
| Base Excess | 0.45 | -0.83 | 1.00 | 0.84 | -0.50 | 0.45 | -0.27 |
| pH | 0.45 | -0.68 | 0.84 | 1.00 | -0.55 | 0.37 | -0.18 |
| PT-INR | -0.37 | 0.54 | -0.50 | -0.55 | 1.00 | -0.41 | 0.14 |
| SBP | 0.28 | -0.47 | 0.45 | 0.37 | -0.41 | 1.00 | -0.25 |
| Heart Rate | -0.10 | 0.31 | -0.27 | -0.18 | 0.14 | -0.25 | 1.00 |

Pairwise Spearman correlation coefficients (complete pairs). Metabolic acidosis markers were strongly inter-correlated (base excess, pH, lactate; |r| up to 0.84), which informed the selection of lactate as the single representative metabolic marker for the multivariate models. Base excess, arterial pH, and PT-INR were excluded to avoid multicollinearity.
