# Supplementary material for: Predictors of mortality available at emergency department arrival in massively transfused trauma patients without severe TBI: A single-center retrospective cohort study
Source: PLoS One. 2026 Oct 1;21(10):e0359963. doi: 10.1371/journal.pone.0359963 (PMC13630199; doi:10.1371/journal.pone.0359963)
Supplement: Supplementary file 4 [file pone.0359963.s004.docx]

**S4 Table.** Variance inflation factors for the sequential multivariate models.

| **Variable** | **Primary Model** | **Secondary Model** | **Exploratory Model** |
| --- | --- | --- | --- |
| GCS | 1.00 | 1.31 | 1.32 |
| Age | 1.01 | 1.06 | 1.08 |
| ISS | 1.01 | 1.03 | 1.13 |
| Lactate | — | 1.38 | 1.47 |
| Time-to-Transfusion | — | — | 1.27 |
| RBC units at 4 h | — | — | 1.33 |

All variance inflation factors were below 2.5, confirming the absence of problematic multicollinearity in the final refined models.
