# Supplementary material for: Predictors of mortality available at emergency department arrival in massively transfused trauma patients without severe TBI: A single-center retrospective cohort study
Source: PLoS One. 2026 Oct 1;21(10):e0359963. doi: 10.1371/journal.pone.0359963 (PMC13630199; doi:10.1371/journal.pone.0359963)
Supplement: Supplementary file 5 [file pone.0359963.s005.docx]

**S5 Table.** Sensitivity analyses: 24-hour mortality and the full cohort including severe traumatic brain injury.

| **Variable / Analysis** | **OR** | **95% CI** | **p-value** |
| --- | --- | --- | --- |
| 24-hour mortality (primary cohort, N = 114) | — | — | — |
| GCS | 0.805 | 0.730–0.889 | <0.001 |
| Age | 0.997 | 0.973–1.021 | 0.798 |
| ISS | 0.976 | 0.926–1.027 | 0.349 |
| AUC = 0.760 | — | — | — |
| Full cohort incl. severe TBI (N = 165) | — | — | — |
| GCS | 0.758 | 0.696–0.825 | <0.001 |
| Age | 1.020 | 1.000–1.041 | 0.053 |
| ISS | 1.001 | 0.966–1.038 | 0.946 |
| AUC = 0.822 | — | — | — |

GCS remained the strongest independent predictor of mortality in both sensitivity analyses. In the full cohort including severe TBI patients, age showed a borderline association, likely reflecting the poor prognosis of elderly patients with severe head injury.
