# Supplementary material for: Predictors of mortality available at emergency department arrival in massively transfused trauma patients without severe TBI: A single-center retrospective cohort study
Source: PLoS One. 2026 Oct 1;21(10):e0359963. doi: 10.1371/journal.pone.0359963 (PMC13630199; doi:10.1371/journal.pone.0359963)
Supplement: Supplementary file 6 [file pone.0359963.s006.docx]

**S6 Table.** Post hoc analysis of prehospital physiology and of the field-to-emergency-department change in vital signs (primary cohort, head AIS < 4, N = 124).

**A. Availability and distribution**

| **Variable** | **Documented, n (%)** | **Measurable, n (%)** | **Survivors, median (IQR)** | **Non-survivors, median (IQR)** | **p** |
| --- | --- | --- | --- | --- | --- |
| Field systolic blood pressure (mmHg) | 106 (85.5) | 103 (83.1) | 80 (70–100) | 80 (70–90) | 0.330 |
| Field heart rate (bpm) | 121 (97.6) | 118 (95.2) | 92 (80–111) | 98 (80–127) | 0.572 |
| Field shock index | — | 103 (83.1) | 1.1 (0.8–1.5) | 1.2 (1.0–1.6) | 0.219 |
| Change in systolic blood pressure, field to ED (mmHg) | — | 75 (60.5) | 0 (−20 to 10) | −10 (−20 to 0) | 0.192 |
| Change in heart rate, field to ED (bpm) | — | 103 (83.1) | 5.5 (−2.0 to 17.2) | 3.0 (−8.0 to 17.0) | 0.366 |

**B. Univariate logistic regression for in-hospital mortality**

| **Variable** | **n** | **OR** | **95% CI** | **p** |
| --- | --- | --- | --- | --- |
| Field systolic blood pressure (per 10 mmHg) | 103 | 0.98 | 0.84–1.14 | 0.778 |
| Field heart rate (per 10 bpm) | 118 | 1.09 | 0.96–1.24 | 0.164 |
| Field shock index (per 0.1) | 103 | 1.06 | 0.97–1.15 | 0.215 |
| Field hypotension (<90 mmHg) | 103 | 1.13 | 0.50–2.52 | 0.774 |
| Field systolic blood pressure unobtainable | 124 | 0.71 | 0.06–8.05 | 0.782 |
| Change in systolic blood pressure, field to ED (per 10 mmHg) | 75 | 0.88 | 0.73–1.05 | 0.153 |
| Change in heart rate, field to ED (per 10 bpm) | 103 | 0.90 | 0.77–1.05 | 0.191 |

**C. Prehospital variables added to the primary model (GCS, age, ISS)**

| **Model** | **n** | **GCS, OR (95% CI)** | **Added variable, OR (95% CI)** | **p** | **LR test p** | **AUC** | **AUC, same patients, primary model** |
| --- | --- | --- | --- | --- | --- | --- | --- |
| Primary + field systolic blood pressure | 101 | 0.77 (0.68–0.86) | 1.005 (0.987–1.023) | 0.608 | 0.610 | 0.792 | 0.780 |
| Primary + change in systolic blood pressure, field to ED | 75 | 0.76 (0.65–0.90) | 0.986 (0.967–1.006) | 0.174 | 0.165 | 0.772 | 0.740 |

Field vital signs are the single set of prehospital measurements documented by emergency medical services and held in the trauma registry; only patients transported directly from the scene were eligible for this study. Field readings of 0 mmHg or 0 bpm were treated as unobtainable and excluded from the continuous analyses, in the same way as unmeasurable readings at emergency department arrival; unobtainable field systolic blood pressure (n = 3) is reported separately as a binary variable. The change from field to emergency department is calculated for patients in whom both readings were measurable. Percentages in panel A are of the 124 patients in the cohort; p values are from the Wilcoxon rank-sum test. In panel C, the likelihood ratio test and the AUC in the last column compare each model with the primary model fitted to exactly the same patients. These analyses were performed after peer review and were not prespecified. AUC, area under the receiver operating characteristic curve; CI, confidence interval; ED, emergency department; GCS, Glasgow Coma Scale; ISS, Injury Severity Score; IQR, interquartile range; LR, likelihood ratio; OR, odds ratio.
