# Supplementary material for: Predictors of mortality available at emergency department arrival in massively transfused trauma patients without severe TBI: A single-center retrospective cohort study
Source: PLoS One. 2026 Oct 1;21(10):e0359963. doi: 10.1371/journal.pone.0359963 (PMC13630199; doi:10.1371/journal.pone.0359963)
Supplement: Supplementary file 7 [file pone.0359963.s007.docx]

# S1 Checklist. STROBE checklist for cohort studies

**Study: Predictors of Mortality Available at Emergency Department Arrival in Massively Transfused Trauma Patients Without Severe TBI: A Single-Center Retrospective Cohort Study**

| **Item No.** | **Section/Topic** | **Recommendation** | **Reported on Page/Section** |
| --- | --- | --- | --- |
| 1 | Title and abstract | (a) Indicate the study's design with a commonly used term in the title or the abstract (b) Provide in the abstract an informative and balanced summary of what was done and what was found | (a) Title and Abstract: the design is named as a single-center retrospective cohort study.  (b) Abstract: unstructured summary of objective, design, cohort, models, principal results and interpretation. |
| **INTRODUCTION** | | | |
| 2 | Background/rationale | Explain the scientific background and rationale for the investigation being reported | Introduction, paragraphs 1–3: hemorrhage as the leading preventable cause of trauma death, existing scores predict the need for massive transfusion rather than mortality within the transfused population. Paragraph 4: prehospital treatment and physiologic trajectory as determinants of the arrival profile. Paragraph 5: severe traumatic brain injury as a competing mortality mechanism. |
| 3 | Objectives | State specific objectives, including any prespecified hypotheses | Introduction, final paragraph: to evaluate predictors of mortality available at emergency department arrival in massively transfused trauma patients after excluding severe traumatic brain injury, and to determine whether bedside parameters alone allow early risk stratification. |
| **METHODS** | | | |
| 4 | Study design | Present key elements of study design early in the paper | Materials and methods, 'Study design and setting': single-center retrospective cohort study. |
| 5 | Setting | Describe the setting, locations, and relevant dates, including periods of recruitment, exposure, follow-up, and data collection | Materials and methods, 'Study design and setting': tertiary referral trauma center, Gwangju, Republic of Korea; study period January 2018 – December 2023; registry and medical record data accessed for research on 16/07/2026. |
| 6 | Participants | (a) Give the eligibility criteria, and the sources and methods of selection of participants (b) For matched studies, give matching criteria and number of exposed and unexposed | (a) Materials and methods, 'Study population': registry-based identification, inclusion criteria (ISS ≥ 9, direct admission, ≥ 5 pRBC units within 4 h or ≥ 10 within 24 h), exclusion of first transfusion after operating-room entry and of head AIS ≥ 4; Fig 1 gives the numbers.  (b) Not applicable — this is a single-cohort study with no matching. |
| 7 | Variables | Clearly define all outcomes, exposures, predictors, potential confounders, and effect modifiers | Materials and methods, 'Data collection' (demographic, injury, arrival physiologic, prehospital, laboratory and transfusion variables) and 'Outcome measures' (in-hospital mortality as primary, 24-hour mortality as secondary). |
| 8 | Data sources/measurement | For each variable of interest, give sources of data and details of methods of assessment | Materials and methods, 'Data collection': institutional trauma registry and electronic medical records; prehospital vital signs documented by emergency medical services. All variables were recorded in routine clinical care. |
| 9 | Bias | Describe any efforts to address potential sources of bias | Materials and methods, 'Data collection': physiologically unobtainable vital-sign readings are treated as missing for continuous analyses and carried by a separate unmeasurable-blood-pressure indicator, so that non-physiologic values do not enter the estimates. 'Statistical analysis': complete-case analysis with the missing pattern reported (S1 and S2 Tables), collinearity assessment before model building. Discussion, 'Limitations': selection bias, residual confounding from mild-to-moderate head injury, and unmeasured treatments (tranexamic acid, intubation, prehospital treatment). |
| 10 | Study size | Explain how the study size was arrived at | Materials and methods, 'Study population': all consecutive patients meeting the criteria during the six-year study period (172 identified, 124 analyzed); no formal sample-size calculation was possible for this design, and the consequences are stated in Discussion, 'Limitations'. |
| 11 | Quantitative variables | Explain how quantitative variables were handled in the analyses | Materials and methods, 'Statistical analysis': continuous variables as median (interquartile range); time-to-first-transfusion additionally grouped into four prespecified categories (≤ 15, 16–30, 31–60, > 60 minutes) for the trend test; prehospital variables scaled per 10 mmHg, per 10 bpm and per 0.1 shock index units in the post hoc analysis. |
| 12 | Statistical methods | (a) Describe all statistical methods (b) Describe any methods used to examine subgroups and interactions (c) Explain how missing data were addressed (d) If applicable, describe analytical methods taking account of sampling strategy (e) Describe any sensitivity analyses | (a) Materials and methods, 'Statistical analysis': Wilcoxon rank-sum and Fisher exact tests, univariate logistic regression, three sequential multivariate logistic models, Cochran–Armitage trend test, AUC and Hosmer–Lemeshow calibration.  (b) Subgroups: the cohort is defined by head AIS < 4 and the full cohort is examined as a sensitivity analysis; no interaction terms were fitted.  (c) Complete-case analysis; missingness reported in S1 Table and mortality of complete versus incomplete cases in S2 Table.  (d) Not applicable — no loss to follow-up for in-hospital mortality.  (e) Sensitivity analyses: full cohort including severe traumatic brain injury (S5 Table) and the post hoc prehospital analysis (S6 Table). |
| **RESULTS** | | | |
| 13 | Participants | (a) Report numbers at each stage of study (b) Give reasons for non-participation at each stage (c) Consider use of a flow diagram | (a) Results, 'Study population' and Fig 1: 172 identified, 48 excluded for head AIS ≥ 4, 124 analyzed.  (b) Reasons for exclusion are given in the same paragraph and in Fig 1.  (c) Fig 1 is the flow diagram. |
| 14 | Descriptive data | (a) Give characteristics of study participants and information on exposures and potential confounders (b) Indicate number of participants with missing data for each variable of interest | (a) Results, 'Baseline characteristics', Table 1 and Fig 2.  (b) Missing values per variable are listed in the Table 1 footnote and in S1 Table.  (c) Not applicable — the outcome is in-hospital mortality with complete ascertainment in the analyzed cohort. |
| 15 | Outcome data | Report numbers of outcome events or summary measures | Results, 'Baseline characteristics': 51 of 124 patients died in hospital (41.1%), 28 within 24 hours; Results, '24-hour mortality': 28 of 118 (23.7%) with 24-hour data available. |
| 16 | Main results | (a) Give unadjusted estimates and, if applicable, confounder-adjusted estimates and their precision (b) Report category boundaries when continuous variables were categorized (c) If relevant, consider translating estimates of relative risk into absolute risk | (a) Unadjusted estimates in Table 2; adjusted estimates for the three sequential models in Table 3 and Fig 3, with the variables entered in each model and the reason for entering them given in Materials and methods, 'Statistical analysis'.  (b) Time-to-first-transfusion is reported both as a continuous variable and in four categories (Results, 'Time-to-first-transfusion and mortality'; Fig 5).  (c) Absolute mortality per time-to-transfusion category is given in Fig 5. |
| 17 | Other analyses | Report other analyses done — e.g., analyses of subgroups and interactions, and sensitivity analyses | Results, 'Prehospital physiology and field-to-ED change in vital signs' (post hoc, S6 Table); '24-hour mortality' (S5 Table); 'Sensitivity analysis' in the full cohort including severe traumatic brain injury (S5 Table); model discrimination compared with GCS alone (Fig 4). |
| **DISCUSSION** | | | |
| 18 | Key results | Summarise key results with reference to study objectives | Discussion, first paragraph: the Glasgow Coma Scale at arrival is the single most informative predictor, and a bedside model centered on it discriminates as well as models adding laboratory and transfusion variables. |
| 19 | Limitations | Discuss limitations of the study, taking into account sources of potential bias or imprecision | Discussion, 'Limitations': single-center retrospective design and selection bias; cohort size and absence of external validation; residual confounding from mild-to-moderate head injury; prehospital treatment, serial field vital signs and field GCS not recorded; tranexamic acid and intubation status unavailable; complete-case analysis with unmeasurable blood pressure in 31%. |
| 20 | Interpretation | Give a cautious overall interpretation of results considering objectives, limitations, multiplicity of analyses, results from similar studies, and other relevant evidence | Discussion, paragraphs 2–7: the interpretation of a depressed Glasgow Coma Scale as a composite physiologic signal, the behavior of prehospital and arrival hemodynamics, the shared information between the metabolic markers and the Glasgow Coma Scale, and the reading of time-to-first-transfusion as confounding by indication rather than a causal effect. |
| 21 | Generalisability | Discuss the generalisability (external validity) of the study results | Discussion, 'Limitations': single-center data from one Korean trauma system and the absence of external validation limit generalizability; Discussion, prehospital paragraph: system-dependent prehospital data capture. Conclusion: external validation is called for. |
| **OTHER INFORMATION** | | | |
| 22 | Funding | Give the source of funding and the role of the funders for the present study | Funding: the authors received no specific funding for this work. |
