# Supplementary material for: Predictors of mortality available at emergency department arrival in massively transfused trauma patients without severe TBI: A single-center retrospective cohort study
Source: PLoS One. 2026 Oct 1;21(10):e0359963. doi: 10.1371/journal.pone.0359963 (PMC13630199; doi:10.1371/journal.pone.0359963)
Supplement: Peer review history

# Peer review: Decision Letter 0

**DOI:** 10.1371/journal.pone.0359963.r001

**Authors:** Moises Auron

14 Sep 2026

Dear Dr. Kim,

Thank you for submitting your manuscript to PLOS One. After careful consideration, we feel that it has merit but does not fully meet PLOS One’s publication criteria as it currently stands. Therefore, we invite you to submit a revised version of the manuscript that addresses the points raised during the review process.

Please submit your revised manuscript by Oct 14 2026 11:59PM. If you will need more time than this to complete your revisions, please reply to this message or contact the journal office at plosone@plos.org. When you're ready to submit your revision, log on to https://www.editorialmanager.com/pone/ and select the 'Submissions Needing Revision' folder to locate your manuscript file.

- A letter that responds to each point raised by the academic editor and reviewer(s). You should upload this letter as a separate file labeled 'Response to Reviewers'.
- A marked-up copy of your manuscript that highlights changes made to the original version. You should upload this as a separate file labeled 'Revised Manuscript with Track Changes'.
- An unmarked version of your revised paper without tracked changes. You should upload this as a separate file labeled 'Manuscript'.

If you would like to make changes to your financial disclosure, please include your updated statement in your cover letter. Guidelines for resubmitting your figure files are available below the reviewer comments at the end of this letter.

If applicable, we recommend that you deposit your laboratory protocols in protocols.io to enhance the reproducibility of your results. Protocols.io assigns your protocol its own identifier (DOI) so that it can be cited independently in the future. For instructions see: https://journals.plos.org/plosone/s/submission-guidelines#loc-laboratory-protocols. Additionally, PLOS One offers an option for publishing peer-reviewed Lab Protocol articles, which describe protocols hosted on protocols.io. Read more information on sharing protocols at https://plos.org/protocols?utm_medium=editorial-email&utm_source=authorletters&utm_campaign=protocols.

As the corresponding author, your ORCID iD is verified in the submission system and will appear in the published article. PLOS supports the use of ORCID, and we encourage all coauthors to register for an ORCID iD and use it as well. Please encourage your coauthors to verify their ORCID iD within the submission system before final acceptance, as unverified ORCID iDs will not appear in the published article. *Only*  the individual author can complete the verification step; PLOS staff *cannot*  verify ORCID iDs on behalf of authors.

We look forward to receiving your revised manuscript.

Kind regards,

Moises Auron, MD, FAAP, FACP, SFHM, FRCP (Lon), FRCPCH

Academic Editor

PLOS One

**Journal Requirements:**

When submitting your revision, we need you to address these additional requirements.

1. Please ensure that your manuscript meets PLOS ONE's style requirements, including those for file naming. The PLOS ONE style templates can be found at

https://journals.plos.org/plosone/s/file?id=wjVg/PLOSOne_formatting_sample_main_body.pdf and

https://journals.plos.org/plosone/s/file?id=ba62/PLOSOne_formatting_sample_title_authors_affiliations.pdf

2. For studies involving third-party data, we encourage authors to share any data specific to their analyses that they can legally distribute. PLOS recognizes, however, that authors may be using third-party data they do not have the rights to share. When third-party data cannot be publicly shared, authors must provide all information necessary for interested researchers to apply to gain access to the data. (https://journals.plos.org/plosone/s/data-availability#loc-acceptable-data-access-restrictions)

For any third-party data that the authors cannot legally distribute, they should include the following information in their Data Availability Statement upon submission:

1) A description of the data set and the third-party source

2) If applicable, verification of permission to use the data set

3) Confirmation of whether the authors received any special privileges in accessing the data that other researchers would not have

4) All necessary contact information others would need to apply to gain access to the data

3. Your ethics statement should only appear in the Methods section of your manuscript. If your ethics statement is written in any section besides the Methods, please delete it from any other section.

4. Please ensure that you refer to Figure 5 in your text as, if accepted, production will need this reference to link the reader to the figure.

5. Please include captions for your Supporting Information files at the end of your manuscript, and update any in-text citations to match accordingly. Please see our Supporting Information guidelines for more information: http://journals.plos.org/plosone/s/supporting-information.

6. If the reviewer comments include a recommendation to cite specific previously published works, please review and evaluate these publications to determine whether they are relevant and should be cited. There is no requirement to cite these works unless the editor has indicated otherwise.

7. Please review your reference list to ensure that it is complete and correct. If you have cited papers that have been retracted, please include the rationale for doing so in the manuscript text, or remove these references and replace them with relevant current references. Any changes to the reference list should be mentioned in the rebuttal letter that accompanies your revised manuscript. If you need to cite a retracted article, indicate the article’s retracted status in the References list and also include a citation and full reference for the retraction notice.

[Note: HTML markup is below. Please do not edit.]

Reviewers' comments:

Reviewer's Responses to Questions

**Comments to the Author**

1. Is the manuscript technically sound, and do the data support the conclusions?

Reviewer #1: Yes

**********

2. Has the statistical analysis been performed appropriately and rigorously?

Reviewer #1: Yes

**********

3. Have the authors made all data underlying the findings in their manuscript fully available?

The PLOS Data policy

Reviewer #1: Yes

**********

4. Is the manuscript presented in an intelligible fashion and written in standard English?

Reviewer #1: Yes

**********

Reviewer #1: Thank you for the opportunity to review this study. The research question is clearly defined, and the methods and results are presented well.

A) The potential contribution of pre-hospital transfusion, fluid administration, Glasgow Coma Scale (GCS), and vital sign trends as predictors is not addressed until the limitations section. I recommend introducing and discussing these factors earlier in the manuscript.

B) The statistical analysis clearly explains how unmeasurable systolic blood pressure (SBP) and heart rate (HR) were handled. I also appreciate that mortality rates were compared between cases with complete data and those with missing data.

C) The limitations section is well written. However, numbering the limitations does not appear necessary and could be omitted for a smoother presentation.

D) The figures and graphs effectively complement the text rather than creating dependence on them for understanding. I appreciated being able to read and evaluate the manuscript without needing to refer constantly to the figures during the initial review

**********

what does this mean?). If published, this will include your full peer review and any attached files.

If you choose “no”, your identity will remain anonymous but your review may still be made public.

**Do you want your identity to be public for this peer review?**  For information about this choice, including consent withdrawal, please see our Privacy Policy

Reviewer #1: **Yes:**  Debbie J Tolich DNP RN

**********

[NOTE: If reviewer comments were submitted as an attachment file, they will be attached to this email and accessible via the submission site. Please log into your account, locate the manuscript record, and check for the action link "View Attachments". If this link does not appear, there are no attachment files.]

To ensure your figures meet our technical requirements, please review our figure guidelines: https://journals.plos.org/plosone/s/figures

You may also use PLOS’s free figure tool, NAAS, to help you prepare publication quality figures: https://journals.plos.org/plosone/s/figures#loc-tools-for-figure-preparation.

NAAS will assess whether your figures meet our technical requirements by comparing each figure against our figure specifications.

# Author comment: Author response to Decision Letter 1

**DOI:** 10.1371/journal.pone.0359963.r002

16 Sep 2026

Response to Reviewers

Manuscript ID: PONE-D-26-35699

Title: Predictors of Mortality Available at Emergency Department Arrival in Massively Transfused Trauma Patients Without Severe TBI: A Single-Center Retrospective Cohort Study

Journal: PLOS ONE

Dear Dr. Auron and Reviewer #1,

Thank you for the constructive assessment of our manuscript and for the opportunity to submit a revised version. We have addressed every journal requirement and every point raised by Reviewer #1. Reviewer comment A prompted more than a change of emphasis: because our trauma registry does hold one set of prehospital vital signs, we were able to analyze the prehospital physiology directly and report it as a new supporting table, rather than only discussing it. The study design, the pre-specified primary analysis, and the conclusions are unchanged; no patient was added or removed, and every previously reported number remains as it was.

Reviewer comments are reproduced in italics, followed by our response. Page and line numbers refer to the clean revised manuscript, which carries continuous line numbering; the marked-up copy shows the same changes.

Journal requirements

1. PLOS ONE style requirements, including file naming.

The manuscript has been reformatted to the PLOS ONE templates. Specifically:

— Section order now follows the template: title, authors and affiliations; Abstract; Introduction; Materials and methods; Results; Discussion; Conclusion; end matter; References; Supporting information captions. Headings are in sentence case.

— The title block was reduced to the elements the template carries (title, short title, authors with superscript affiliation markers, affiliation, corresponding author e-mail, equal-contribution footnote). Degrees, ORCID iDs, the telephone number and the word/figure/table counts were removed from the manuscript file; all of them are held in the submission system.

— Figures are now cited in the abbreviated form ("Fig 1") and in ascending numeric order at first appearance, and each figure caption has been placed in the text immediately after the paragraph in which the figure is first cited. Ascending order required renumbering two figures; the mapping is given under requirement 4 below.

— Table 1, Table 2 and Table 3 were already in the manuscript file but were grouped in a "Tables" section after the references. Each has been moved to sit directly after the paragraph in which it is first cited, with its caption above it, and the separate "Tables" and "Figure legends" sections have been removed.

— Supporting information items are named in the PLOS format (S1 Table … S6 Table, S1 Checklist); their captions are listed at the end of the manuscript and the in-text citations were changed accordingly (for example, "Supplementary Table S3" is now "S3 Table").

— The figure files were re-exported to the PLOS ONE technical specification (TIFF, LZW compression, 300 dpi, 2250 pixels or fewer across). Three of the five files had been wider than the 2250-pixel limit. Fig 2 was re-laid out from three columns by two rows to two columns by three so that its six panels fit the 7.5-inch width at the original type size; the panels, the data and the statistics in it are unchanged.

— Files are named Manuscript.docx, Revised_Manuscript_with_Track_Changes.docx, Response_to_Reviewers.docx, Fig1.tif–Fig5.tif, S1_Table.docx–S6_Table.docx and S1_Checklist.docx.

Location in the revised manuscript: Throughout. Table 1 now begins on page 11, Table 2 on page 14 and Table 3 on page 15, each directly after the paragraph that cites it; the figure captions are on pages 11 (Fig 1), 13 (Fig 2), 16 (Fig 3) and 17 (Fig 4 and Fig 5).

2. Third-party data and the Data Availability Statement.

The data are patient-level records owned by Chonnam National University Hospital, which we are not permitted to distribute. The Data Availability Statement has been rewritten so that it states the four required elements — what the data are and whose they are, the permission under which we used them, the absence of any special access privilege, and the contact point for other researchers. The same text has been entered in the submission system. It now reads:

"All data underlying the findings of this study are patient-level clinical records held in the trauma registry and electronic medical record system of Chonnam National University Hospital (CNUH), Gwangju, Republic of Korea; the authors do not own these data. The authors were permitted to access and analyze them under approval CNUH-2026-252 from the CNUH Institutional Review Board (IRB), which also granted a waiver of informed consent, and the data were accessed for research purposes on 16/07/2026. Because the records contain potentially identifying and sensitive information about individual trauma patients, ethical and legal restrictions on the public sharing of patient-level clinical data prevent the authors from distributing the dataset. The authors received no special access privileges that would not be available to other qualified researchers who satisfy the same institutional and ethical requirements. Researchers who meet the criteria for access to confidential data may apply to the Institutional Review Board of Chonnam National University Hospital (e-mail: cnuhirb@gmail.com; telephone: +82-62-220-5257)."

Location in the revised manuscript: Data availability, pages 24–25, lines 466–478.

We have also corrected the institutional contact details, which were incomplete in the submitted version; the address and telephone number above are those of the CNUH Institutional Review Board office that handles data-access applications.

3. Ethics statement in the Methods only.

The "Ethics Statement" paragraph that appeared in the end matter of the submitted manuscript has been deleted. The ethics information now appears only in Materials and methods, under "Study design and setting", where the sentence on the Declaration of Helsinki was merged so that nothing was lost:

"The study was approved by the Institutional Review Board of Chonnam National University Hospital (IRB No. CNUH-2026-252) and was conducted in accordance with the Declaration of Helsinki. The requirement for informed consent was waived due to the retrospective nature of the study."

Location in the revised manuscript: Materials and methods, "Study design and setting", page 6, lines 86–95.

4. Reference to Figure 5 in the text.

Thank you for catching this. The boxplot figure was indeed never cited. It is now cited in Results, under "Baseline characteristics": "The distributions of these variables by survival status are shown in Fig 2." Because PLOS ONE requires figures to be numbered in the order of first citation, citing this figure where it belongs also required two figures to change number. The mapping is:

— Fig 1 (study flow diagram) — unchanged, cited in Materials and methods and in Results.

— Fig 5 in the submitted version (boxplots of key variables) is now Fig 2, cited in Results, "Baseline characteristics".

— Fig 2 in the submitted version (forest plot of the primary model) is now Fig 3, cited in Results, "Multivariate analysis".

— Fig 4 (ROC curves) — unchanged, cited in Results, "Multivariate analysis".

— Fig 3 in the submitted version (mortality by time-to-first-transfusion) is now Fig 5, cited in Results, "Time-to-first-transfusion and mortality".

The figure files have been renamed to match, and every figure is now cited at least once, in ascending order. No figure content was altered.

Location in the revised manuscript: Results, "Baseline characteristics", page 13: citation at lines 221–227, caption at lines 228–234.

5. Supporting information captions and in-text citations.

A "Supporting information" section has been added at the end of the manuscript with a caption for every item, and each item is uploaded as its own file:

— S1 Table. Missing data summary.

— S2 Table. Complete-case availability by model.

— S3 Table. Spearman correlation matrix of key predictor variables.

— S4 Table. Variance inflation factors for the sequential multivariate models.

— S5 Table. Sensitivity analyses: 24-hour mortality and the full cohort including severe traumatic brain injury.

— S6 Table. Post hoc analysis of prehospital physiology and of the field-to-emergency-department change in vital signs. (New in this revision, in response to Reviewer comment A.)

— S1 Checklist. STROBE checklist for reporting this retrospective cohort study.

All in-text citations were converted to the PLOS format, and the order in which the supporting items are first cited is now ascending; this required moving the paragraph on missing data and complete-case analysis ahead of the paragraph on variable selection within Statistical analysis. No content was changed in S1–S5 Tables.

Location in the revised manuscript: "Supporting information", page 29, lines 548–556.

6. Citations recommended by the reviewer.

Reviewer #1 did not recommend any specific publication for citation, and we have therefore added no references on that basis.

7. Reference list, including retracted articles.

We re-verified all 18 references against PubMed on 16 September 2026, checking author, journal, year, volume, issue and pages for each, and checking the publication type and correction notices of each record. All 18 are correctly cited, and none is retracted or carries an expression of concern. No changes were made to the reference list, and no reference was added or removed in this revision.

Additional correction found during this revision.

While preparing the supporting files we found that the STROBE checklist submitted with the original manuscript had been carried over from an earlier study by our group on a universal type-O red cell protocol: its "reported on" entries described a before-and-after design with propensity-score matching and a different cohort, neither of which applies to this manuscript. The checklist has been rewritten item by item against the present manuscript and is uploaded as S1 Checklist. No text in the manuscript itself was affected, and we apologize for the oversight.

Re-running every reported number against the analysis dataset during this revision also surfaced one transcription error: the correlation among the metabolic markers was given in the text as "|r| = 0.67–0.84", whereas the value in S3 Table and in the data is 0.68 (lactate versus arterial pH). The text now reads 0.68–0.84. No other reported number changed, and no result or conclusion is affected.

Reviewer #1

We thank Reviewer #1 for the careful and generous reading of the manuscript.

Comment A.

"The potential contribution of pre-hospital transfusion, fluid administration, Glasgow Coma Scale (GCS), and vital sign trends as predictors is not addressed until the limitations section. I recommend introducing and discussing these factors earlier in the manuscript."

We agree, and this comment led to the most substantive change in the revision. Rather than moving the existing sentence forward, we asked what the registry could actually tell us. Because only patients transported directly from the scene were eligible, one set of field vital signs — systolic and diastolic blood pressure and heart rate, documented by emergency medical services — was potentially available for every patient, and the paired field and ED readings allow the change between the scene and hospital arrival to be computed. Serial prehospital measurements, prehospital GCS, and the volumes of fluid and blood products given before arrival are not captured by the registry. We have therefore analyzed what is available and stated plainly what is not, and we have done so from the Introduction onward rather than in the limitations. Five changes were made:

— Introduction: a new paragraph (fourth) explains that the physiology recorded at ED arrival is shaped by prehospital treatment and by the trajectory preceding arrival, and states what our registry does and does not record.

— Materials and methods, "Data collection": a new paragraph describes the prehospital variables, their availability, and the derivation of the field-to-ED change in systolic blood pressure and heart rate.

— Materials and methods, "Statistical analysis": the post hoc prehospital analysis is specified, including the treatment of field readings of 0 mmHg or 0 bpm as unobtainable, consistent with how unmeasurable ED readings were handled in the original analysis.

— Results: a new subsection, "Prehospital physiology and field-to-ED change in vital signs", reports the analysis, with the full detail in S6 Table.

— Discussion: a new paragraph (third) discusses prehospital care and physiologic trajectory as determinants of the arrival profile, what our data show, and what remains unmeasured. The limitations section now carries only the residual gap and refers back to that paragraph.

The findings are as follows. A field systolic blood pressure was documented in 106 of 124 patients (85.5%) and was measurable in 103 (83.1%); a field heart rate was documented in 121 (97.6%) and measurable in 118 (95.2%). A measurable field systolic pressure was therefore available in more patients than a measurable pressure at ED arrival (103 vs. 85). None of the prehospital measures was associated with in-hospital mortality: field systolic blood pressure (OR = 0.98 per 10 mmHg, 95% CI 0.84–1.14, p = 0.778), field heart rate (OR = 1.09 per 10 bpm, 95% CI 0.96–1.24, p = 0.164), field shock index (OR = 1.06 per 0.1, 95% CI 0.97–1.15, p = 0.215) and field hypotension below 90 mmHg (OR = 1.13, 95% CI 0.50–2.52, p = 0.774). The field-to-ED change was likewise unassociated with mortality, for systolic blood pressure (OR = 0.88 per 10 mmHg, 95% CI 0.73–1.05, p = 0.153, n = 75) and for heart rate (OR = 0.90 per 10 bpm, 95% CI 0.77–1.05, p = 0.191, n = 103). Adding field systolic blood pressure to the primary model left GCS unchanged (OR = 0.77, 95% CI 0.68–0.86, p < 0.001) and improved neither fit (likelihood ratio p = 0.610) nor discrimination relative to the same model fitted to the same 101 patients (AUC 0.792 vs. 0.780).

This is consistent with what the ED hemodynamic variables did in the same patients, and it strengthens rather than qualifies the main message: in patients already committed to massive transfusion, blood pressure and heart rate — in the field, at arrival, or as the change between them — discriminate far less well than the level of consciousness. We have been explicit that these analyses were performed after peer review, were not prespecified, and are exploratory; they are reported as supporting information and the primary analysis is unchanged.

Location in the revised manuscript: Introduction, pages 4–5, lines 61–70; Materials and methods, "Data collection", page 7, lines 117–125, and "Statistical analysis", page 10, lines 175–185; Results, "Prehospital physiology and field-to-ED change in vital signs", page 18, lines 313–331; Discussion, pages 20–21, lines 366–384; S6 Table.

Comment B.

"The statistical analysis clearly explains how unmeasurable systolic blood pressure (SBP) and heart rate (HR) were handled. I also appreciate that mortality rates were compared between cases with complete data and those with missing data."

Thank you. We have kept that treatment unchanged and applied the same rule to the prehospital readings added in this revision: a field value of 0 mmHg or 0 bpm is an unobtainable reading rather than a measurement, so it was excluded from the continuous analyses and reported separately as a binary variable (S6 Table). Within Statistical analysis, the paragraph on missing data and complete-case analysis has been moved ahead of the paragraph on variable selection; its content is unchanged, and the move also puts the supporting tables into ascending citation order as PLOS ONE requires.

Location in the revised manuscript: Materials and methods, "Statistical analysis", page 8, lines 141–146.

Comment C.

"The limitations section is well written. However, numbering the limitations does not appear necessary and could be omitted for a smoother presentation."

Agreed, and done. The enumeration ("First … Second … Sixth … Finally") has been removed and the section now reads as continuous prose, beginning: "This study has several limitations. Its single-center, retrospective design limits generalizability and is susceptible to selection bias. The cohort of 124 patients, while typical for single-center massive transfusion series, limits the number of variables that can be entered reliably into multivariate models …". While rewriting, we grouped the absence of external validation with the sample-size limitation, where it belongs, and reduced the prehospital limitation to what remains genuinely unknown, since the rest is now analyzed in the Results. Limitations is also now a subsection heading within the Discussion, so that the reader can find it without the numbering as a signpost.

Location in the revised manuscript: Discussion, "Limitations", pages 23–24, lines 434–452.

Comment D.

"The figures and graphs effectively complement the text rather than creating dependence on them for understanding. I appreciated being able to read and evaluate the manuscript without needing to refer constantly to the figures during the initial review."

Thank you; we have tried to preserve this. The figure content is unchanged, and the new prehospital results are given in full in the text so that they can be read without consulting S6 Table. The one structural change is that the three main tables now sit inside the manuscript, next to the paragraphs that cite them, as the PLOS ONE template requires; their content is unchanged.

Summary

The revision adds one supporting table (S6) and a short Results subsection, adds one paragraph each to the Introduction and the Discussion, moves the ethics statement into the Methods, rewrites the Data Availability Statement and the limitations paragraph, and reformats the manuscript to the PLOS ONE template, including figure renumbering to citation order. The cohort, the pre-specified primary analysis, the reference list and the conclusions are unchanged. We hope the manuscript is now suitable for publication in PLOS ONE, and we thank the editor and Reviewer #1 for their time.

Sincerely,

Hyo-Sin Kim, MD, on behalf of all authors

Department of Surgery, Chonnam National University Medical School and Hospital, Gwangju, Republic of Korea

Attachment Submitted filename: Response_to_Reviewers.docx

# Editor report: Decision Letter 1

**DOI:** 10.1371/journal.pone.0359963.r003

**Authors:** Moises Auron

21 Sep 2026

<p>Predictors of Mortality Available at Emergency Department Arrival in Massively Transfused Trauma Patients Without Severe TBI: A Single-Center Retrospective Cohort Study

PONE-D-26-35699R1

Dear Dr. Kim,

We’re pleased to inform you that your manuscript has been judged scientifically suitable for publication and will be formally accepted for publication once it meets all outstanding technical requirements.

Within one week, you’ll receive an e-mail detailing the required amendments. When these have been addressed, you’ll receive a formal acceptance letter and your manuscript will be scheduled for publication.

An invoice will be generated when your article is formally accepted. Please note, if your institution has a publishing partnership with PLOS and your article meets the relevant criteria, all or part of your publication costs will be covered. Please make sure your user information is up-to-date by logging into Editorial Manager at Editorial Manager® and clicking the ‘Update My Information' link at the top of the page. For questions related to billing, please contact billing support.

If your institution or institutions have a press office, please notify them about your upcoming paper to help maximize its impact. If they’ll be preparing press materials, please inform our press team as soon as possible -- no later than 48 hours after receiving the formal acceptance. Your manuscript will remain under strict press embargo until 2 pm Eastern Time on the date of publication. For more information, please contact onepress@plos.org.

Kind regards,

Moises Auron, MD, FAAP, FACP, SFHM, FRCP (Lon), FRCPCH

Academic Editor

PLOS One

Additional Editor Comments (optional):

Reviewers' comments:

# Editor report: Acceptance letter

**DOI:** 10.1371/journal.pone.0359963.r004

**Authors:** Moises Auron

PONE-D-26-35699R1

PLOS One

Dear Dr. Kim,

I'm pleased to inform you that your manuscript has been deemed suitable for publication in PLOS One. Congratulations! Your manuscript is now being handed over to our production team.

At this stage, our production department will prepare your paper for publication. This includes ensuring the following:

* All references, tables, and figures are properly cited

* All relevant supporting information is included in the manuscript submission,

* There are no issues that prevent the paper from being properly typeset

You will receive further instructions from the production team, including instructions on how to review your proof when it is ready. Please keep in mind that we are working through a large volume of accepted articles, so please give us a few days to review your paper and let you know the next and final steps.

Lastly, if your institution or institutions have a press office, please let them know about your upcoming paper now to help maximize its impact. If they'll be preparing press materials, please inform our press team within the next 48 hours. Your manuscript will remain under strict press embargo until 2 pm Eastern Time on the date of publication. For more information, please contact onepress@plos.org.

You will receive an invoice from PLOS for your publication fee after your manuscript has reached the completed accept phase. If you receive an email requesting payment before acceptance or for any other service, this may be a phishing scheme. Learn how to identify phishing emails and protect your accounts at https://explore.plos.org/phishing.

If we can help with anything else, please email us at customercare@plos.org.

Thank you for submitting your work to PLOS One and supporting open access.

Kind regards,

PLOS One Editorial Office Staff

on behalf of

Dr. Moises Auron

Academic Editor

PLOS One
